# Development of an Emotion-Sensitive mHealth Approach for Mood-State Recognition in Bipolar Disorder

**DOI:** 10.2196/14267

**Published:** 2020-07-03

**Authors:** Henning Daus, Timon Bloecher, Ronny Egeler, Richard De Klerk, Wilhelm Stork, Matthias Backenstrass

**Affiliations:** 1 Institute of Clinical Psychology Centre for Mental Health Klinikum Stuttgart Stuttgart Germany; 2 Faculty of Science Eberhard-Karls-University Tübingen Tübingen Germany; 3 Embedded Systems and Sensors Engineering Research Center for Information Technology Karlsruhe Germany; 4 Sikom Software GmbH Heidelberg Germany; 5 ITK Engineering GmbH Ruelzheim Germany; 6 Institute for Information Processing Technologies Karlsruhe Institute of Technology Karlsruhe Germany; 7 Department of Clinical Psychology and Psychotherapy Institute of Psychology Ruprecht-Karls-University Heidelberg Heidelberg Germany

**Keywords:** bipolar disorder, mood recognition, emotion recognition, monitoring, mobile apps, assistance system, mHealth

## Abstract

Internet- and mobile-based approaches have become increasingly significant to psychological research in the field of bipolar disorders. While research suggests that emotional aspects of bipolar disorders are substantially related to the social and global functioning or the suicidality of patients, these aspects have so far not sufficiently been considered within the context of mobile-based disease management approaches. As a multiprofessional research team, we have developed a new and emotion-sensitive assistance system, which we have adapted to the needs of patients with bipolar disorder. Next to the analysis of self-assessments, third-party assessments, and sensor data, the new assistance system analyzes audio and video data of these patients regarding their emotional content or the presence of emotional cues. In this viewpoint, we describe the theoretical and technological basis of our emotion-sensitive approach and do not present empirical data or a proof of concept. To our knowledge, the new assistance system incorporates the first mobile-based approach to analyze emotional expressions of patients with bipolar disorder. As a next step, the validity and feasibility of our emotion-sensitive approach must be evaluated. In the future, it might benefit diagnostic, prognostic, or even therapeutic purposes and complement existing systems with the help of new and intuitive interaction models.

## Introduction

With a prevalence of more than 1%, bipolar disorder is one of the most common mental disorders worldwide [1]. The disease is associated with the suffering of the affected people and their relatives and poses great challenges to them in their everyday lives [2,3]. The depressive and (hypo) manic episodes can have extensive social and economic consequences for patients with bipolar disorder and their families [3]. In particular, the high relapse rates within bipolar disorder are unsettling for all parties concerned: Even with pharmacological treatment [4] and different psychological approaches [5,6], these relapses cannot completely be prevented in many cases.

Because of the frequently severe and chronic course and the individual and social consequences, additional strategies and support options within patient care are necessary [6,7]. With the proceeding technological development and the digitalization of the health care system, increasing attention has recently been paid to internet- and mobile-based interventions in the field of bipolar disorders [8-10]. Internet-based interventions, such as psychoeducational tutorials, can help to reach a great number of patients. Mobile-based approaches often assess real-time information about illness activity or deliver time-sensitive messages to patients in an ambulatory setting. To achieve this, most systems use smartphone technology, external sensor systems, or wearable devices (portable computer systems that assess and analyze psychophysiological data). In our research project, we developed a new mobile-based assistance system for bipolar disorder. In this viewpoint, we describe the theoretical and technological basis of our approach.

Over the past years, the use of smartphone apps or mobile programs has been investigated with samples of patients with bipolar disorder with an often good feasibility [11-16]. Because of the mobility of digital systems, for example, ambulatory self-assessments can easily be integrated in the patients’ daily routines. This benefits a better availability and can increase the adherence compared with nondigital approaches [11,12,17-19]. Furthermore, the self-assessment approach can be expanded by additional assessments of sensor data: wearable devices or internal smartphone sensors can be used to trace a patient’s mood state [20-22]. Thus, sensor data can aid in automatic recognition of mood-state changes and can support relapse prevention [23-27]. In order to improve disease management as well as treatment compliance and medication adherence in patients with bipolar disorder, self-assessments of patients can be combined with automatic feedback within certain situations [13,14,16,28]. Even simple SMS text message reminders two times per week can improve medication adherence of these patients and help them to create a more positive attitude toward their medication [29]. Interestingly enough, smartphone apps can also support the biological and social rhythms of patients with bipolar disorder. This might lead to a smaller degree of rhythmic disbalances in the long-term course of their disease [30-32]. Beyond that, several studies indicate that mobile-based approaches can reduce the symptom severity in bipolar or other mood disorders [13,14,33-36].

However, the existing approaches neglect the emotional aspects of bipolar disorders. For example, during mood episodes there are typical patterns of experienced emotions: whereas manic states are often characterized by increased happiness or anger and fear, depressive states often show patterns of elevated sadness and disgust [37]. Bipolar disorders are further associated with a generally amplified emotionality [38,39] and difficulties in emotion processing and regulation [40-44], in emotion recognition [45-47], and in the expression of emotions [48,49]. These deficits might partially be related to the current mood state of patients [41,46,47]. Yet, they strongly affect their social and global functioning and are related to severe outcome variables such as suicidality [39,40,42-45,48]. Consequently, emotional aspects have a great impact on the patients’ everyday lives and the long-term course of bipolar disorder.

So far, mobile-based approaches have analyzed the keyboard activity of patients with bipolar disorder [22] or even ambient sound samples [31,32,50] or voice features during phone calls [23,51]. However, to our knowledge, none of the referenced approaches have analyzed the emotional content of audio data or social interactions. Moreover, psychological research has so far focused on emotional responses of fully or partially remitted patients with bipolar disorder by analyzing their facial expressions during standardized tasks [48,49]. Yet, there does not exist any mobile-based approach that analyzes facial expressions of these patients regarding their emotional cues. In reference to the importance of emotional aspects in bipolar disorder, they should play a more important role in the design of mobile Health (mHealth) approaches too. Compared with other behavioral measures, the emotional expressions of patients with bipolar disorder could reflect their emotional reactivity more sensitively [49]. Beyond that, the ambulatory setting would allow to monitor individual changes over time and mood states in real life [52,53]. Thus, emotion-sensitive mHealth systems for bipolar disorder might even increase our understanding of the experienced and expressed emotions of patients or of their impact on the patients’ social and global functioning.

## The EmAsIn Project

Within the *EmAsIn* project (Emotion-sensitive Assistance systems for the reactive psychological Interaction with people) we developed the first emotion-sensitive, technical assistance system for patients with bipolar disorder. Because self-assessments of symptoms are the well-established basis of mood monitoring in bipolar disorder [11,12,17-19], our system also includes regular self-assessments of patients. It further analyzes automatically assessed sensor data, because physiological or behavioral data have been shown to be useful in mood-recognition approaches [23-27], and sleep data have been in the focus of bipolar research for a certain period now [54]. In addition, we incorporated third-party assessments of relatives or related parties, because some patients themselves emphasize the importance of an external point of view regarding their current condition [55]. As a consequence, some of the pressure might be taken off the constant self-monitoring of patients with bipolar disorder. The additionally assessed data could also help in individual cases or during certain periods (eg, during severe mood episodes) with less reliable or accurate self-assessments [56,57]. The importance of emotional aspects of bipolar disorders [37-49] motivated us to develop the key component of our system, the emotion-sensitive *Story of the Day* module. It analyzes audio and video data to explore the emotional experiences and expressions of patients. While many apps in this field are poorly investigated [58], we emphasized the importance of an empirically validated basis of our emotion-sensitive approach [59-61]. To consider the patients’ point of view, we initially started a dialogue with patients with bipolar disorder, which indicated their overall positive attitudes toward our innovative ideas [55].

## System Concept and Features

Our assistance system includes an Android smartphone app and a connected wearable device, which can be both code protected and password protected. It uses multichannel data acquisition to realize an early recognition of mood-state changes in bipolar disorder. It further intends to complement the rather technical exchange of information between systems and patients with new and intuitive interaction models. Therefore, it aims to recognize socioemotional cues in human communication behavior and hereby infer conclusions about emotional and mental states. To this end, the emotion-sensitive Story of the Day module analyzes the verbal and facial expressions of patients in short and actively user-triggered recordings with respect to their emotional content or the presence of emotional cues. Consequently, this module collects active and passive emotion-related data of patients with bipolar disorder and relies on its regular use (see “Story of the Day” section). If all the features of the assistance system are activated, it can gather information about mood states and the course of bipolar disorders with the aid of the following resources:

- daily self-assessments of patients regarding their mood, activity level, and other relevant symptoms;
- regular third-party assessments by relatives or other related parties regarding the most important symptoms;
- automatic assessments of (psycho-) physiological parameters such as heart rate or resting heart rate;
- automatic assessments of sleep duration and quality;
- automatic assessments of several behavioral parameters such as recognized activities, movement/acceleration, steps per day, range of motion, or smartphone usage behavior (eg, used apps, number of calls per day);
- assessments of auditive information (eg, voice, emotional content, speech duration, or breaks) as emotional cues and indicators of mood states;
- assessments of visual information (facial expressions) as emotional cues and indicators of mood states.

All data resources are presented in Table 1, which also indicates their mandatory or optional usage within the assistance system. Users can switch between different features and tasks by opening the menu of the app. If this feature is activated, the app reminds them of their tasks by using push notifications at a predefined time of the day. Daily self-assessments consist of six 7-point items (from –3 to 3) about symptoms that are relevant to depressive as well as to (hypo-) manic mood states. Negative values are predominantly associated with depressive symptoms, whereas positive values should reflect (hypo) manic states. In addition, as in earlier approaches [19], each user can choose from a given list of potential early warning signs (like *mixed emotions* or *increased caffeine intake*) or can create new items. These items are then incorporated into the daily self-assessments, where they are evaluated with *yes* or *no.* The third-party assessments are very similar to the self-assessments, but they are realized by using a separate and individually secured web application.

The assistance system uses smartphone sensors to assess several of the behavioral aspects, for instance, with regard to movement or social interaction (without analyzing content information). Information about sleeping behavior and (psycho-) physiological data is continuously collected with the help of the connected wearable device, which users wear on their wrists (see Multimedia Appendix 1 for more detailed information). Whereas most of the sensor data are automatically assessed, users are asked to use the Story of the Day module on a regular basis (eg, once per day). Once information is gathered through the different sources, the assistance system integrates all data with the aid of an external server and visualizes the accessed information in the form of graphic representations over time. In addition, users can implement a digital version of their own, personal crisis plan with individual strategies for different mood states and locally stored contact information. They can also enter information about their actual medication to use the medication reminder of the system. To facilitate the handling, users can use their own and secured web application to insert and manage information.

**Figure 1:**
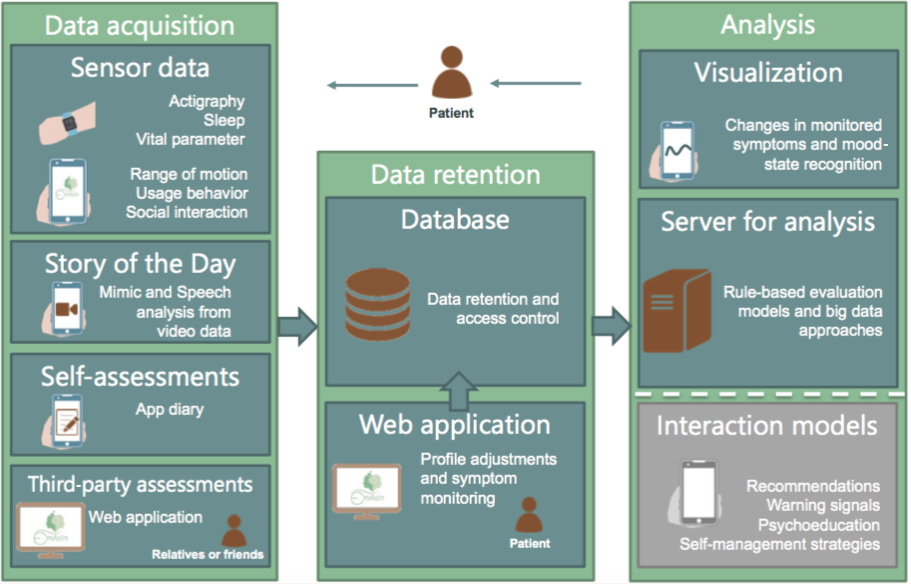
Concept of the assistance system.

The system is supposed to recognize mood-state changes in patients with bipolar and to react by sending warning signals or, like other approaches [13,14,28,30], by proposing recommendations (eg, to consult a doctor) and self-management strategies. All system components are fully developed; only the interventions that depend on the automatic mood-state recognition have not been implemented at the actual stage of development. Apart from long-term analyses using big data approaches, neural networks, and machine learning approaches [62], we are pursuing rule-based evaluation models to allow for an increasing accuracy of the state recognition. To this end, patients can adjust the importance of certain parameters for their own mood-recognition approach. For example, they can assign values between 1 and 3 to each relevant factor (self-assessments, third-party assessments, behavioral, physiological and sleep data, or emotional expressions) to implicate their individual importance (with 1 being *less important*, 2 *moderately important*, and 3 *very important*). The system then includes the individual assignments when integrating and analyzing the assessed data. Beyond that, patients may also assign these values to the warning signs, which are then analyzed as separate factors. Figure 1 illustrates the concept of the assistance system.

**Table 1:** Data resources of the assistance system.

Information source and its components			Parameters	Category
**Sensor data**				
	**Smartphone**			
		Location	Range of motion^a^, visited locations^a^	Activity and behavior
		Accelerometer	Movements/acceleration^a^	Activity and behavior
		Smartphone usage	Usage duration^a^, number of calls^a^, click rate^a^	Activity and behavior
		Social interaction	Usage of social apps^a^, number of messages (SMS text messages, emails, instant messengers)^a^	Social behavior
	**Wearable**			
		Vital	Heart rate^a^, resting heart rate^a^	Physiological data
		Movement patterns	Steps/distance per day^a^, recognized activities^a^	Activity and behavior
		Sleep	Sleeping/wake up time^b^	Sleep duration
			Bedtime/getting out of bed^b^	Sleep efficiency
			Wake phases^b^, activity at night^b^	Sleep quality
**Self-assessments**				
	**Smartphone**			
		Diary	Self-assessments^b^	Self-image
**Third-party assessments**				
	**Web application**			
		Diary	Third-party assessments^a^	Perception by others
**Story of the Day**				
	**Smartphone**			
		Microphone	Speech duration^b^, breaks^b^, words per minute^b^	Activity/urge to speak
			Emotional words^b^, color of the voice^b^, loudness^b^	Emotional expression
		Camera	Facial expressions^b^	Emotional expression

^a^Optional.

^b^Mandatory.

## Story of the Day

As opposed to earlier approaches, which analyzed ambient sound samples or voice features without processing emotional information [23,31,32,50,51], our emotion-sensitive module analyzes intentionally recorded sequences regarding the contained auditive and visual emotional cues. When a recording is initiated on the start screen of the Story of the Day module, the app uses the smartphone camera to capture video data. In order to secure a sufficient recording quality, the users mount their smartphones in well-positioned holders before activating this feature. Furthermore, external microphones are attached to the smartphones to improve the audio quality of the recordings. At the beginning of each recording sequence, the users are asked to describe an important event of their day. After telling their story the recording must actively be ended and the users are asked if they want to save the recording. If the microphone and camera do not record any information (ie, no recognized voice or face), the recording is automatically discontinued.

The app analyzes the assessed auditive and visual information separately. The verbal information is analyzed regarding the use of emotional words, the color of the voice, its energy level (ie, loudness), the verbal fluency, and the speech rate as well as the extent to which the story is narrated. The count of emotional words in automatic transcriptions of the used language of each recording is based on the *Linguistic Inquiry and Word Count* (LIWC) program [59] and includes the emotional categories of positive emotions, negative emotions, sadness, anxiety, or anger. The voice analysis follows the *EmoVoice* approach [60], a framework that uses acoustic signals as emotional classification units and recognizes emotional or mental states on the basis of these signals. For each audio file, the system analyzes segments of 250 ms and assigns values between 0 and 1 to the categories anger, boredom, disgust, fear, happiness, and sadness. The automatic recognition of emotions in facial expressions during the Story of the Day recordings is based on the *Facial Action Coding System* (FACS) [61]. In short intervals of 1 frame/second, facial expressions are examined evaluating the 4 emotions, namely, happiness, sadness, anger, and anxiety. For each emotion, the percentage frequency of its coding is calculated.

## Discussion

Internet- and mobile-based approaches have become increasingly important to psychological research in the field of bipolar disorders. In particular, the aspiring mHealth approach benefits a consistent self-monitoring of patients with bipolar disorder [11,12,17-19] and allows for mood-recognition approaches based on automatically assessed sensor data [20,21,23-27]. Our new assistance system incorporates some of the well-known components of mHealth systems for bipolar disorder and combines them with the innovative features of third-party assessments and the analysis of emotional expressions.

While the self-perception of patients with bipolar disorder is certainly the most important factor in mood monitoring, self-assessments can be less reliable in specific cases or during severe episodes [56,57]. Beyond that, some patients trust the assessments of relatives or related parties more than their own perception, when it comes to their mood states [55]. Thus, our third-party assessments could help to gain a more comprehensive view regarding the patients’ mood states. In reference to the great burden, which bipolar disorders are putting on the relationships of patients [63], the third-party assessments might even reduce some of the tension: They can shift the external feedback from possibly strained direct interactions to regular web-based assessments.

Our Story of the Day module, as far as we know, is the first mobile-based approach to analyze the emotional expressions of patients with bipolar disorder. As opposed to the analysis of ambient sound samples or voice features during phone calls [23,31,32,50,51], the actively user-triggered Story of the Day recordings allow us to analyze visual and auditive information as well as the emotional content of the spoken language. The well-established FACS [61], LIWC [59], and EmoVoice approach [60] should provide the technical implementation of our emotion-recognition approach with some helpful framework. This development is especially promising when the effects of emotional deficits on the social and global functioning of patients are considered [39,40,42-45,48]. Consequently, our emotion-sensitive approach is not only interesting in the context of mood-state recognition but might also increase our understanding of experienced and expressed emotions of patients with bipolar disorder. The received feedback in regard to their emotional expressions might be especially informative to patients without regular or with strained social interactions. Moreover, the emotional and narrative character of our Story of the Day module might aid a less technical or distant usage experience and might motivate patients to reflect upon their daily (social) experiences and interactions.

Of course, our new assistance system comes with its limitations. Most importantly, the predictive value of our approach concerning its mood-state recognition and its efficacy and effectiveness with respect to relapse prevention has to be addressed in empirical studies with patients with bipolar disorder. In addition, not all patients approve of the involvement of relatives or related parties in their mood-monitoring approach [55]. Our Story of the Day module must also be used on a regular basis to enable its automatic analysis of emotional expressions. Thus, like self-monitoring systems, our emotion-sensitive approach may depend on the patients’ mood state and motivation. However, as a consequence, the Story of the Day module does not automatically assess audio or video data and thus does not interfere with the patients’ privacy or personal space. Beyond that, our assistance system allows patients to activate or deactivate certain features (eg, the third-party assessments) and meets the patients’ expectations of flexible systems [55,64]. Furthermore, based on our preliminary findings, we estimate that the Story of the Day recordings should not take up more than 2 minutes per day. In the future, our Story of the Day approach might be even less effortful as it could possibly be realized in a more natural setting without smartphone holders or external microphones.

Whereas the EmoVoice approach [60] and, in part, the LIWC approach [59] incorporate the analysis of verbally expressed disgust into our emotion-sensitive module, the Story of the Day module does not recognize this emotion in the facial expressions of patients. Because disgust is one of the more frequently experienced emotions in bipolar disorder [37], subsequent mobile-based FACS approaches [61] should possibly be programmed to include this emotion as well. Finally, our Story of the Day module does not react to suicidal statements and suicidality is not assessed during the self-assessments. The monitoring of suicidal tendencies or even time-sensitive interventions in case of severe suicidal crises with technological help comes with extensive ethical or legal considerations and can have unexpected effects [65]. Accordingly, before implementing such features into mobile-based approaches for bipolar disorder, their feasibility and effects should be examined thoroughly.

With this in mind, there are still some issues to be dealt with in the further development of our assistance system and more research is needed to examine the clinical value of our system. However, our assistance system and its new and innovative features might improve the understanding of the patients’ mood state and could provide important information about the patients’ expressed emotions as well as their (social) interaction behavior. Considering the strong association between emotional aspects and the social and global functioning of patients with bipolar disorder, in the future, emotion-sensitive systems might be even useful during emotion-based treatment approaches in bipolar disorder [66-68].

## Conclusion

The mHealth approach offers many opportunities to support patients with bipolar disorder in their everyday struggle with their disease. However, the existing mobile-based approaches do not consider the importance of emotional aspects in bipolar disorder and their implications regarding the social and global functioning of patients. With our assistance system, we aim to address this issue and have therefore implemented the emotion-sensitive Story of the Day module. With the help of this module, our system analyzes the emotional experiences and expressions of patients besides regular self-assessments and third-party assessments as well as the analysis of further sensor data. In the future, emotion-sensitive approaches might not only benefit a better understanding of the patients’ emotional states, but they might also be used to complement the technical exchange of information between systems and patients with more intuitive interaction models. Moreover, they might even support emotion-based interventions in bipolar disorder.

## Appendix

Multimedia Appendix 1 Assessment and analysis of sleep data.

## Abbreviations

FACS: Facial Action Coding System
LIWC: Linguistic Inquiry and Word Count
