# Supplementary material for: Development of an Emotion-Sensitive mHealth Approach for Mood-State Recognition in Bipolar Disorder
Source: JMIR Ment Health. 2020 Jul 3;7(7):e14267. doi: 10.2196/14267 (PMC7367525; doi:10.2196/14267)
Supplement: Multimedia Appendix 1 [file mental_v7i7e14267_app1.docx]

**Further detail regarding the assessment and analysis of sleep data**

Our assistance system for bipolar disorder incudes well-established wearable devices, which estimate sleep patterns according to changes in heart rate (heart rate variability) as well as movement patterns during the night. Beyond that, patients can add self-assessment data regarding their sleep time, their sleep duration and their estimated time needed to fall asleep. Thus, the system allows for an individual comparison of objective and subjective sleep data.
